# Modeling and Testing of Flexible Structures with Selected Planar Patterns Used in Biomedical Applications

**DOI:** 10.3390/ma14010140

**Published:** 2020-12-30

**Authors:** Pavel Marsalek, Martin Sotola, David Rybansky, Vojtech Repa, Radim Halama, Martin Fusek, Jiri Prokop

**Affiliations:** 1 Deparment of Applied Mechanics, Faculty of Mechanical Engineering, VŠB—Technical University of Ostrava, 17. listopadu 2172/15, 708 00 Ostrava, Czech Republic; martin.sotola@vsb.cz (M.S.); david.rybansky@vsb.cz (D.R.); vojtech.repa.st@vsb.cz (V.R.); radim.halama@vsb.cz (R.H.); martin.fusek@vsb.cz (M.F.); 2 Department of Surgical Studies, Faculty of Medicine, University of Ostrava, Dvorakova 7, 701 03 Ostrava, Czech Republic; jiri.prokop@fno.cz; 3 Department of Surgery, University Hospital Ostrava, 17. listopadu 1790/5, 708 00 Ostrava, Czech Republic

**Keywords:** wearable, flexible, structure, stiffness, biomedical, mechanics, simulation, pattern, 3D print, PA12

## Abstract

Flexible structures (FS) are thin shells with a pattern of holes. The stiffness of the structure in the normal direction is reduced by the shape of gaps rather than by the choice of the material based on mechanical properties such as Young’s modulus. This paper presents virtual prototyping of 3D printed flexible structures with selected planar patterns using laboratory testing and computer modeling. The objective of this work is to develop a non-linear computational model evaluating the structure’s stiffness and its experimental verification; in addition, we aimed to identify the best of the proposed patterns with respect to its stiffness: load-bearing capacity ratio. Following validation, the validated computational model is used for a parametric study of selected patterns. Nylon—Polyamide 12—was chosen for the purposes of this study as an appropriate flexible material suitable for 3D printing. At the end of the work, a computational model of the selected structure with modeling of load-bearing capacity is presented. The obtained results can be used in the design of external biomedical applications such as orthoses, prostheses, cranial remoulding helmets padding, or a new type of adaptive cushions. This paper is an extension of the conference paper: “Modeling and Testing of 3D Printed Flexible Structures with Three-pointed Star Pattern Used in Biomedical Applications” by authors Repa et al.

## 1. Introduction

Simple shapes have been connected to form patterns since ancient times. Mostly, such tasks had only an aesthetic function, such as the decoration of exterior surfaces. The process of covering the surface with a pattern is called tesselation and the geometric shapes forming the pattern are called tiles. Tesselation in two dimensions (2D), also called planar tiling, is a process of arranging the tiles to fill a surface according to predefined rules. Periodic tiling has a repeating pattern. Patterns are common also in nature—for instance, an almost perfect pattern of hexagonal cells can be found in honeycombs [1,2]. Recent studies showed the potential of patterns in designing structures as an emerging solution for the reduction of the product weight, consumption of energy during production, and of manufacturing time [3,4]. For example, a lattice structure is often recommended to maintain the stiffness of structures while reducing weight (and, thus, material consumption) [5,6]. An application of an aluminum honeycomb structure can be found in the article by Phu et al. [7] investigating the impact properties of such a solution in impact attenuators, requiring a strictly defined crushing behavior.

The requirements for controlling the stiffness of structures are becoming more common in biomedical applications. Therefore, the authors of this work focused on the design of flexible structures (FS) that can be produced by means of additive manufacturing, especially by 3D printing. The term FS typically describes a thin shell with a pattern of holes decreasing the structure’s stiffness in the normal direction. This approach allows the stiffness and other mechanical properties of the part to be controlled by the shape of the empty areas (holes) such as the size of the gaps, structure thickness, curvature, etc., rather than by the choice of material. Typical applications of FS include the flexible parts of orthoses or prostheses that are in direct contact with the patient’s body [8], cranial remoulding helmets padding [4], new types of adaptive cushions and others [9]. In this paper, authors focused mainly on external biomedical application (for example, already mention helmet padding). However, it should be noted that some FS are used in vivo, such as flexible micro-LEDs for optogenetic neuromodulation [10] or flexible films (patches) for energy harvesting [11].

Parts with such a flexible structure can be designed with desired deformation behavior. For example, Schumacher et al. [12] were studying mechanical properties of structured sheet materials. They managed to establish a link between the complex deformation behavior and elastic properties through numerical methods (homogenization). Doing so, they calculated various structures and evaluated their Young’s modulus, Poisson’s ratio and bending stiffness, which helped them determine if the structures had isotropic or anisotropic behavior.

Bickel et al. [13] also studied the deformation behavior of flexible structures. They aimed to optimize the base material structure to achieve desired deformation behavior. For this purpose, they used the finite element method (FEM) as the design tool, combined with additive manufacturing. Lastly, it is possible to design aesthetic modifications of parts that also preserve their structural integrity; see e.g., various papers by Schumacher et al. [14]. Those authors presented a novel method to design shells factoring at the same time the aesthetics of the product, its stability and material efficiency into the design through structural optimization.

Wearable FS applications must strictly meet the stiffness requirements established through medical examination because they must be adapted to the user body. One of the aims of the use of flexible structures is to reduce swelling and to allow a proper flow of body fluids. The use of holed design also facilitates breathing through the skin. Due to the enormous advantages of 3D printing methods such as Selective Laser Sintering (SLS) or Multi-Jet Fusion (MJF), the complex shape of FS can be printed without supports, which allows the production of the final shape without additional machining. SLS is based on gradual sintering of powder layers using a laser beam, offering high accuracy even with complex shapes [15]. The procedure is simple: A thin layer of powder is applied on the working surface, heating the material slightly below the sintering temperature. Next, the laser beam heats the exact locations of the area, which leads to sintering. After sintering, the base plate is moved down by the thickness of a single layer and the whole process is repeated. The base plate and work chamber are heated, which prevents the part from warping or shrinking during printing. MJF is similar to SLS but after the powder layer is applied, an additional fusion agent must be jetted with precision on the exact locations to be sintered. Then, a high-power infrared (IR) light passes over the bed and the areas containing the fusion agent reach the sintering temperature while other areas remain untouched. This method is significantly faster and, in some cases, more accurate than SLS [16]. However, the color of the product, which is usually grey to black due to the use of IR light, represents (for some applications, at least), a possible disadvantage.

The objective of this work was to prepare a method for determining the stiffness of an isohedral flexible structure using planar patterns. However, as performing all experiments with custom-printed structures would be extremely expensive, a computer model using the Finite element method (FEM) was created for evaluation of the structures’ stiffness. This approach allowed a great improvement in cost-effectiveness as only a tensile test of the material used for printing was necessary to acquire the input for the model. The stiffness derived from the model was then compared with experimental results. This approach does not aim to fully remove the experiments but to significantly decrease their number. After preparing the computer model, an additional parametric study was performed to demonstrate the advantages of this approach. The preliminary results of the work using a linear computational model were presented at the EAN 2020 conference and published in the book of extended abstracts by authors Repa et al. [17].

## 2. Materials and Methods

**Figure 1:**
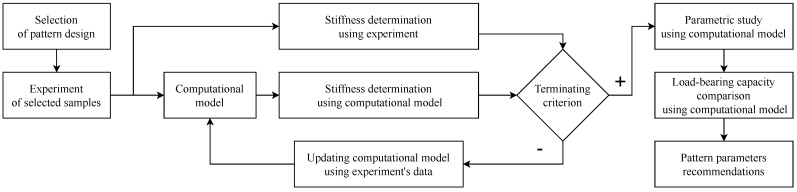
A diagram showing the research workflow of modeling and testing of flexible structures.

The proposed methods are divided into four stages. The first stage describes the design of the experiment, aiming at the selection of patterns suitable for the specimens. Moreover, the choice of the material of the specimens is discussed in the first stage. Necessary experiments evaluating the stiffness of the structure are also performed at this stage. The remaining stages focus on the design of the test specimens and testing device. In the second stage, the design of a simplified non-linear computational model replacing extensive experiments is described. The result of the second stage is a validated model that sufficiently describes the experiment in terms of structural stiffness. In the third stage, the geometric properties of the selected patterns are analyzed using a parametric study. Since the designed specimens are sensitive to failure when subject to large deformations, a full non-linear computer model of the selected pattern is designed in the last stage, used for comparison of load-bearing capacity. In this way, it is possible to assess not only the stiffness of the structure but also its load-bearing capacity. Figure 1 shows a diagram summarizing the workflow described in the previous paragraph.

### 2.1. First Stage: Experimental Design

#### 2.1.1. Selection of Pattern Design

In this work, the starting patterns were selected based on the authors’ experience with designing biomedical applications.

**Figure 2:**
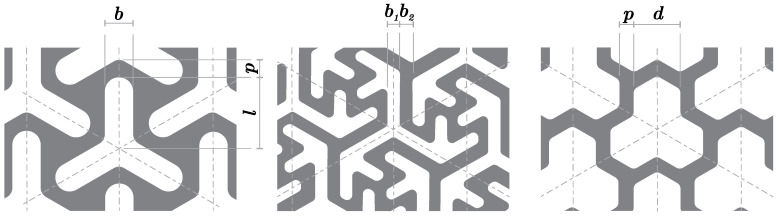
Selected pattern designs for flexible structures—three-pointed star pattern A (**left**), modified three-pointed star pattern B (**middle**) and tri-hexagonal pattern C (**right**).

Three types of FS pattern were used, namely the three-pointed star pattern (A), modified three-pointed star pattern (B) and tri-hexagonal pattern (C). All patterns were created by isohedral tesselation and prepared based on custom-selected input parameters, which are detailed in Figure 2. The three-pointed star (A) and tri-hexagonal (C) patterns have a three-axis reflection symmetry while the modified three-pointed star pattern (B) has a three-fold rotation symmetry; this, according to Schumacher et al., [12] means that their behavior during loading should be isotropic.

#### 2.1.2. Material Selection

**Figure 3:**
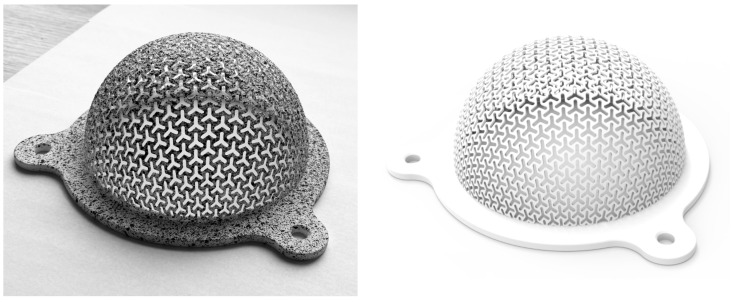
Printed specimen A with a spot pattern for DIC method (**left**) and visualization of geometric model (**right**).

The complex geometry of FS is difficult to be manufactured using conservative ways, see Figure 3. Additive manufacturing (AM), on the other hand, offers freedom in designing complex geometries. Nevertheless, not all AM methods are suitable for FS printing. The methods that can be used for their production should offer support-free printing. Suitable methods are mentioned in the Introduction.

For the purposes of this study, Nylon (Polyamide 12, PA12), was chosen as an appropriate material suitable for 3D printing due to its material properties described in the article by Marsalek et al. [4]. PA12 is a thermoplastic material offering good chemical and wear resistance and high toughness. It has a great strength to weight ratio and is also recommended for biomedical applications [18]. 3D printed products usually have anisotropic behavior dependent on the orientation of printing. However, the SLS printing method used in our case study allowed us to neglect the variation of Young’s modulus [19]. The initial elastic material properties of PA12 are detailed in Table 1; they were obtained from the article by Stoia et al. [20] (in that article, PA12 is labeled as PA2200). Obtained values were for printed samples with the orientation angle of 90 degrees. The table also contains the yield strength and tangent modulus of the PA12 material to describe the plastic behavior of the specimen for computer modeling of designed experiment.

#### 2.1.3. Description of Testing Methodology

The experimental verification of the shell stiffness was inspired by the laboratory testing of cranial orthoses published by Marsalek et al. [4].

**Figure 4:**
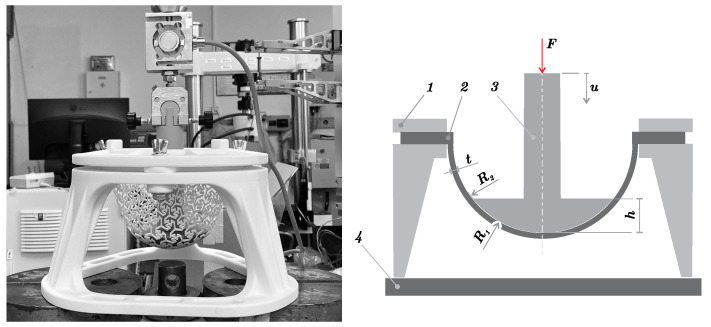
The experimental equipment (**left**) and its scheme (**right**): 1—fastening system, 2—specimen, 3—plunger, 4—frame.

The authors of the article used a plunger to gradually deform the structure and analyze the displacement. Also, the authors used a segment of a large radius sphere as a testing specimen in their paper. In the new testing methodology proposed in the presented paper, the specimen representing the FS is a hemisphere with a fixed inner radius of $R_{1}\;=\;65.0$ mm, see the scheme in Figure 4. We expect the new specimen shape to be suitable for a wider range of applications due to type of loading. The hemisphere shape was subjected to two types of loading. The area near the plunger was subjected mainly to bending while the area near the rim was subjected to a combination of bending and tensile load. The curvature of the hemisphere was also closer to applications such as helmet padding (almost hemispheric shape) or orthoses and prostheses (almost cylindrical shape). A fastening system was designed for clamping specimens and a plunger with dimensions of $R_{2}\;=\;65.0$ mm and h=18.0 mm was prepared for loading the specimens. The continuous load was generated and measured using a universal testing machine TESTOMETRIC 50050CT (Testometic, Rochdale, UK). The fastening system was designed using the CAD software (SW) Autodesk Inventor (Autodesk, San Rafael, CA, USA). Every component of the testing device, including samples, was made by additive manufacturing. Most of them were printed using the SLS method and PA12 material. Only the plunger was printed using the Fused Filament Fabrication (FFF) method and PLA (polylactic acid) material. It must be emphasized that specimens were more compliant than the fastening system and plunger. The fastening system was designed with robust legs with stiffeners. Also, both the fastening system and plunger were printed as full material (i.e., without using lattice infilling), which further increased their stiffness.

Samples were designed using a combination of two CAD SW, Rhinoceros (Robert McNeel & Associates, Seattle, WA, USA, RHN) and Ansys Spaceclaim (Ansys, Canonsburg, PA, USA, SCDM). Most of the common CAD software solutions tend to deform the shape of the structure when applied on curved surfaces. However, CAD RHN offers robust modeling of such complex structures even on non-planar surfaces. SCDM supports the use of custom-made scripts for creating such structures on the reference surface. At the same time, SCDM supports easy export of the geometric model representation (CAD) into ANSYS Workbench (Ansys, Canonsburg, United States of America, AWB). The designed specimens (3D printed and virtual one) using pattern A are shown in Figure 3.

Altogether, four specimens were printed for the purposes of the experiment, namely two specimens marked A1 with a three-pointed star pattern employing parameters *b* = 2.00 mm, *l* = 5.00 mm, and *p* = 2.35 mm, and two specimens with a modified three-pointed star pattern marked B1 with parameters of $b_{1}$ = 1.75 mm, and $b_{2}$ = 1.75 mm, see Figure 2. Both A1 and B1 specimens were printed with two thicknesses (*t* = 1.00 and 1.25 mm, respectively). The parameters of these patterns are listed in Table 2.

### 2.2. Second Stage: Virtual Modeling of Structural Stiffness

**Figure 5:**
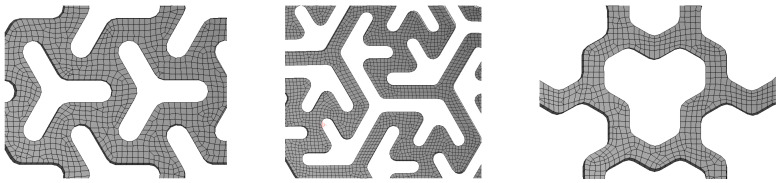
Visualization of the detail of the finite element mesh (specimen A—**left**, specimen B—**middle** and specimen C—**right**).

To be able to replace the FS experimental measurements with a virtual one, two different computational models were designed. These static computational models were solved using FEM in SW AWB. The spatial geometry of the samples was converted into a shell structure and the specimen rim was removed. A surface body for discretization was obtained as the inner surface of the specimen. Shell discretization (elements SHELL181) was used to describe the behavior of the structure. The thickness of elements was set to be outwards. The basic characteristics of the shell elements in the application are described in [21]. The number of finite elements and nodes used for models is described in the Table 3. The discretization is shown in Figure 5.

The first simplified non-linear model was used for determining structural stiffness. The simplified model did not contain plunger. The second full non-linear model was used for comparison of the load-bearing capacity. Analyses of both models took into account large deformation and displacement. In both models, a bilinear PA12 material elastic-plastic was used for describing plastic behavior. The mechanical properties for both material models are listed in Table 1. It is necessary to mention that modeling the load-bearing capacity of structures is very difficult as it is necessary to consider the complexity of problem using an iterative solver with an unsymmetric Newton Raphson option. This topic was studied previously by the group of Horyl and Marsalek [22,23].

**Figure 6:**
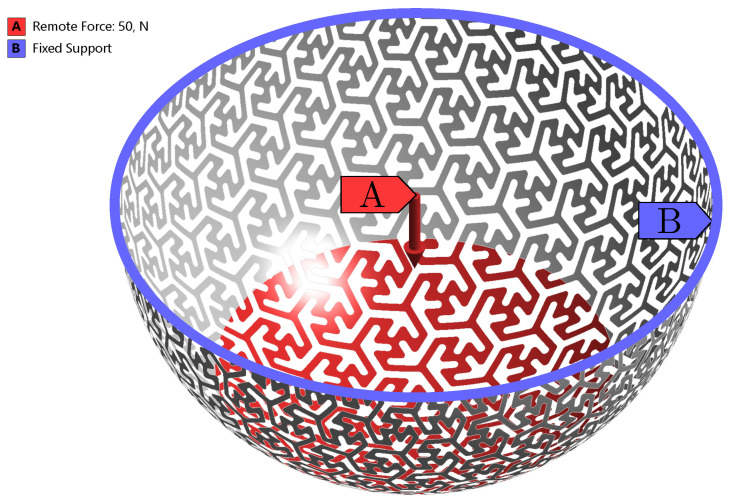
Boundary conditions for a simplified non-linear model analyses of structure stiffness.

**Figure 7:**
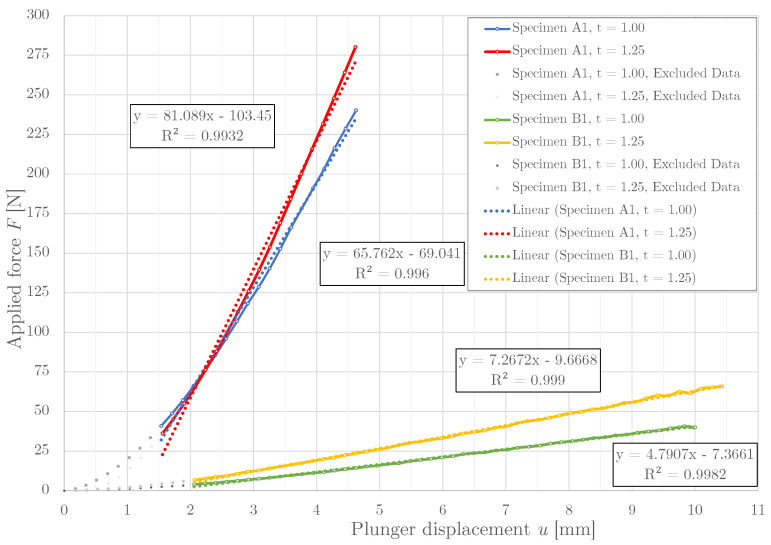
The relationship between the applied force *F* and measured plunger displacement *u*.

The simplified non-linear computational model for prediction of the structure stiffness used only a simplified structure of the individual specimens A, B and C (without the rim), see Figure 6. Since the plunger was much stiffer than the tested structure, the boundary condition corresponding to the specimen loading (by plunger) was defined by using an ANSYS function Remote force with deformable behavior (red color). Remote force is placing remote (pilot) node that is connected to the part using multi-point connection, which can be set as deformable or rigid behavior. Rigid behavior of the remote force is not appropriate due to significant alteration of stiffness in the area of application. Using this function, the force acting on the remote point is distributed to the applied area. The static load was gradually increased. The outer part of the structure (where the rim was removed) was fixed (blue color), all degrees of freedom (three displacement and three rotations) were set to 0 mm, respectively 0 rad. The friction between the plunger and the specimen was disregarded. The load on the structure was gradually increased until the established value of the loading force was reached. In the case of specimen A, the maximal load was F=250 N, in the case of specimen B, the maximal load was F=50 N. Specified values of the loading forces correspond to the experiment, see Figure 7. For the last type of specimens (specimen C), the maximal load was F=150 N.

Due to the 3D printer capabilities and printing tolerances, the resulting thicknesses of the printed specimens were lower than required. As a result, we had to adjust the models to the real thicknesses, i.e., the $t_{r}$ values were reduced by 0.20 mm during stiffness modeling.

### 2.3. Third Stage: Parametric Study

All selected patterns were used for the parametric study of the FS stiffness employing the validated simplified computer model. In all, the stiffness was evaluated for 54 combinations of the pattern and real thickness. For each of the three pattern types (3-point star, modified three-point star, tri-hexagonal), three variants of the pattern geometry were analyzed, see Table 4 for individual parameters. In addition, five thicknesses for each of these pattern geometries were evaluated, ranging from 0.80 mm to 2.05 mm with the step size of 0.25 mm.

### 2.4. Fourth Stage: Modeling of the Load-Bearing Capacity

**Figure 8:**
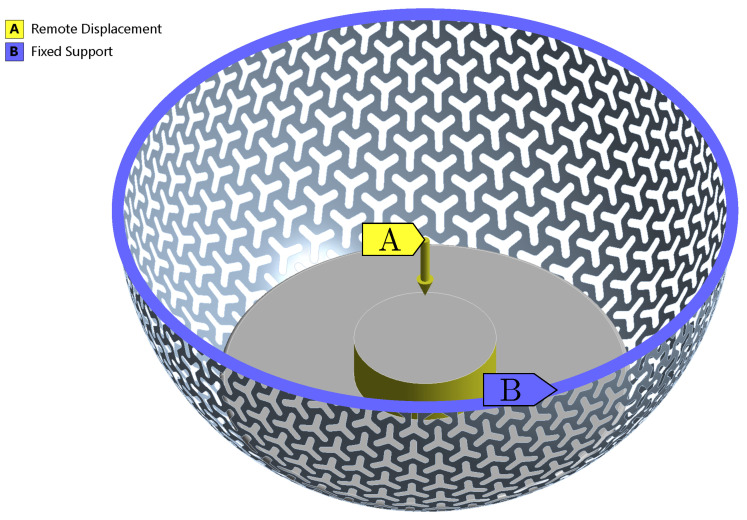
Boundary conditions for a full non-linear model analyses of load-bearing capacity.

To capture the physical behavior of the specimen a full non-linear computational model was created. The computational model was based on a parametric geometrical model of the specimen with a three-point star pattern, just like in the case of simulation for stiffness determination. In this case, however, the computational model was complemented by a spatial geometrical model of the plunger. The plunger was considered rigid, as its stiffness is significantly higher than that of the specimens. Also, the Augmented Lagrange contact algorithm using friction between the specimen and plunger was considered. The friction coefficient $f_{s}$ = 0.30 was obtained from the article by Bai et al. [24] focusing on tribological and mechanical properties of PA12. The gradually increasing displacement was applied on the plunger, up to the value of $u_{max}\;=\;4.00$ mm. The boundary conditions for the non-linear model are depicted in Figure 8.

## 3. Results

Experiments were performed on 4 specimens (A1 and B1 in two thickness variants) and their results were used for validation of the computational model. This model was in turn used for a parametric stiffness study that was performed for various parameters of patterns A, B, and C. Based on the results, the pattern type combining the best properties (best stiffness:load-bearing capacity), namely pattern A, was selected for the load-bearing capacity modeling.

### 3.1. Stiffness Determination Using Experiment

The measured relationship between the applied force *F* and the plunger displacement *u* is depicted in Figure 7. It is apparent that the relation between the force *F* and displacement is non-linear at the beginning. This was caused by adjusting the backlash (gap) between the plunger and the specimen. After adjustment, it can be viewed as an almost linear dependence. This meant that the beginning had to be excluded from the dataset. The aim of the data exclusion was to obtain a linear regression with a coefficient of determination $R^{2}$ of > 0.99. The resulting stiffness values are listed in Table 5.

### 3.2. Stiffness Determination Using the Computational Model

**Figure 9:**
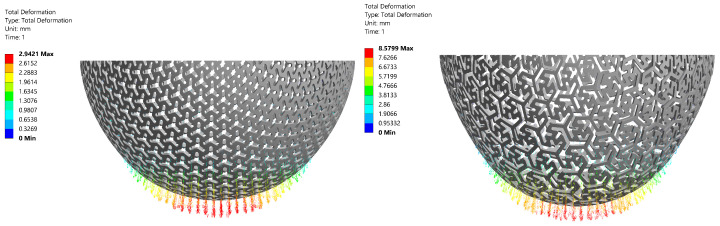
The vectordisplacement fields corresponding to the applied force *F* = 250 N for Specimen A1 (**left**), respectively the applied force *F* = 50 N for specimen B1 (**right**), real thickness $t_{r}$ = 1.05 mm.

**Figure 10:**
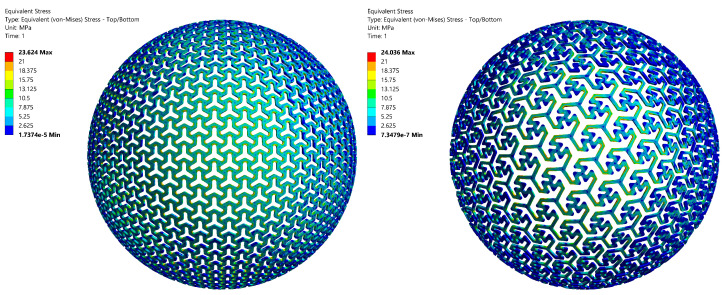
Von Mises stress fields corresponding to the applied force *F* = 250 N for Specimen A1 (**left**), respectively the applied force *F* = 50 N for specimen B1 (**right**), real thickness $t_{r}$ = 1.05 mm.

The validated computational model calculated the relationship between the applied force *F* and maximal displacement *u*, from which stiffness was determined by linear approximation (in the same way as in the case of experimental measurement). A high agreement between the experiment and simulation was achieved (see Table 6). The vector displacement field and Von Mises stress field corresponding to the loading forces of *F* = 250 N and *F* = 50 N, respectively, are shown in Figure 9 and Figure 10. It can be seen that Von Mises Stress is higher than the considered yield strength $\sigma_{Y}$ = 21 MPa at local areas (near the notch, depicted by red color). Plastic deformation occurs in these areas.

### 3.3. Stiffness Determination Using a Parametric Study

**Figure 11:**
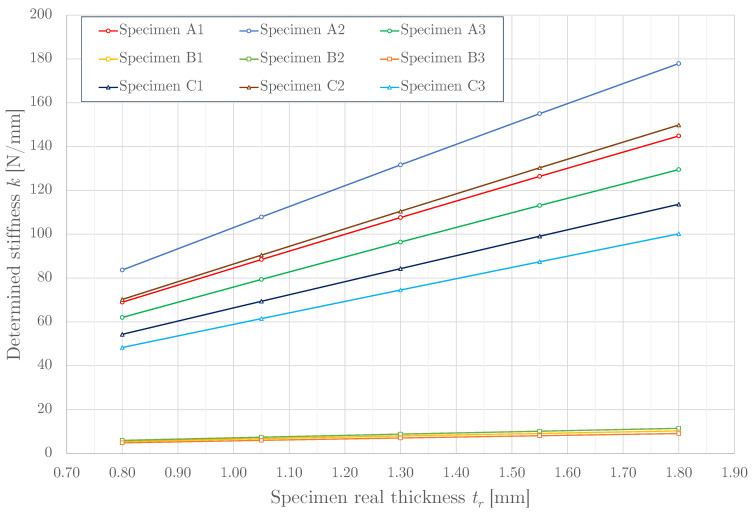
The relationship between the determined stiffness *k* and specimen real thickness $t_{r}$.

Obtained results are listed in Table 7. It shows that the influence of the stiffness *k* grows with increasing the real thickness $t_{r}$. Figure 11 displays mentioned results in the graphical form. Results show that stiffness can be controlled by parameters of the gaps. For example, increasing gaps parameters *b* and *l* by 0.25 mm (this was difference between specimen A1 and A3) reduced stiffness approximately by 10%.

Table 8 shows the influence of the thickness demonstrated on the pattern A. It is apparent that the FS stiffness almost linearly grew with the real thickness in the range of $t_{r}$ = 0.80–2.05 mm. Thanks to this growth, which can be described by linear regression with coefficient of determination $R^{2}$ > 0.99, see Figure 11, stiffness can be easily controlled by changing thickness.

### 3.4. Comparison of the Load-Bearing Capacity

**Figure 12:**
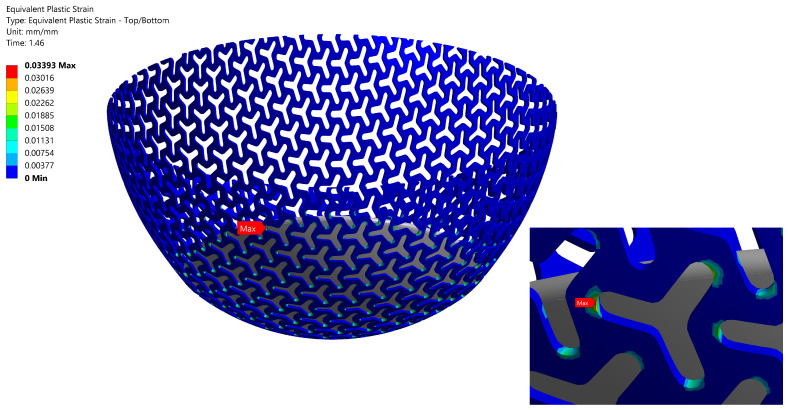
The plastic strain field corresponding to the failure load $F_{max}$ = 276 N for specimen A1, real thickness $t_{r}$ = 0.80 mm.

To design the computational model, we destroyed the specimen A1 with a real thickness of $t_{r}$ = 0.80 mm (after evaluating stiffness data) to obtain the value of the load-bearing capacity. The maximal failure load of the FS $F_{max}\;=\;276$ N was measured in the experiment. This force was important for estimating the value of the plastic strain $\varepsilon_{pb}$ = 0.0339, which was obtained using full non-linear model (with contact) after subjecting specimen A1 to the maximal failure load $F_{max}$, see Table 9. The plastic strain field corresponding to the failure load force $F_{max}$ N with details on the critical area is shown in Figure 12. Plastic deformation occurred locally on the borders of the pattern empty area. A critical area with the highest plastic strain was near the edge of the plunger, where additional bending of FS acted. This is also the area of the breakage of the specimen during the initial experiment.

After calculating the value of plastic strain and locations, authors were able to compare the pattern types A2 and A3 with A1. A failure load was also calculated for specimens A2 and A3 with the real thickness of $t_{r}$ = 0.80 mm. The highest plastic strain on specimen A2 and A3 occurred in the area similar to that of the specimen A1. The load-bearing capacity values corresponding to all calculated failure loads are listed in Table 10. It should be noted that only one specimen was destroyed and values in Table 10 cannot be viewed as statistically correct; still, it gave us some approximate values of the load-bearing capacity of all geometries.

## 4. Discussion

In this paper, two ways of determining the stiffness of 3D printed flexible structures (FS) are presented. The first way was the construction of a novel testing device replacing the traditional tensile and flexural testing techniques used for testing of internal structure properties [25,26]. The second way is a custom-developed validated computational model based on the Finite element method (FEM). It was also demonstrated that the computational model is suitable for the description of the FS behavior and, as such, could be used to evaluate the FS stiffness. The agreement between the initial experiment and simulation was achieved and the model was subsequently used for a parametric study with three different FS pattern types, each of them with three variations of pattern geometries and five thickness variations (Figure 11).

Even though all testing devices were 3D printed, they were stiffer than tested specimens. This influence was analyzed by finite element analysis of the fastening system. Maximal deformation of the fastening system (in the direction of force) was only *u* = 0.11 mm corresponds to load force *F* = 250 N. Deformation of the fastening system did not significantly affect performed experiments (compared to deformations of specimens). Therefore, the influence of the fastening system stiffness could be neglected. For measuring the specimens with higher thickness, a new robust fastening system is necessary to design.

The proposed experimental technique was found to be an important complementary tool in the design of the 3D printed FS with a three-point star pattern by non-linear computational modeling. Moreover, a computational model for analyzing the FS load-bearing capacity was presented, yielding results with satisfactory accuracy compared to the real, experimentally verified, failure load. Such a non-linear computational model can be used for prediction of the FS load-bearing capacity and for prevention of specimen breakage during testing. One should not forget the possibility of plastic strains occurring in small curvatures at higher load forces. It should be noted that comparing linear and non-linear computational models, the latter one describing laboratory experiment closely.

This paper presents two different computational models, both solving a static structural problem with elasto-plastic behavior defined by bilinear material model. In the first model for stiffness determination, we used a simplified non-linear model without a plunger. For precise evaluation of the load-bearing capacity, however, a non-linear model with a plunger had to be constructed. Although the material model provided satisfactory results, it could be substituted with an even better one, such as Chaboch material model, if even more accurate results were necessary. Nevertheless, the main advantage of the presented non-linear material model is its simplicity, needing only two input variables.

It should be noted that the thicknesses of the computational models were corrected to fit the real printed ones and, therefore, they were comparable with experiments. The deviation from the planned specimen thickness occurred during printing. This might have been caused by the low thickness of the shell to which a lower than required amount of the Nylon powder may have adhered. Measurement was performed on various places of the specimen using the vernier caliper. In some areas, the thickness was reduced by 0.18 mm, sometimes by 0.22 mm. The thickness of the computational model was reduced on average by 0.20 mm. To increase the accuracy of the thickness, it would be necessary to use a very accurate 3D scanner and to subsequently insert the acquired point cloud into the calculation.

Assuming the isotropic material behavior could be viewed as an incorrect premise since printed materials do not behave the same in all directions. The material model should be constructed for orthotropic or, even better, anisotropic behavior. Some results from the measurement suggest a possibility of creep and viscoelastic behavior under load.

During the experiment, strain and displacement were also measured by the DIC method; however, obtained data were not satisfying. The measurement was limited by the used template size, which had to be set to “large” to obtain some data. Another complication was the presence of a “holed” curved surface, which caused difficulties in detecting the pattern on the outer surface. All these problems meant that although it was possible to correctly assess the displacement, the strain/stress measurement could not be properly evaluated. In future experiments, therefore, the use of the DIC method should be redesigned to prevent the mentioned problems. Successful use of this method on a curved surface is described in a paper by Halama et al. [27].

Although the measurement using the DIC method was not successful, it sparked a few ideas on how to improve the procedure. First, background should be added to the inner surface to create contrast with the outer pattern. Second, some post-manufacturing process should be applied to ensure that the outer surface does not remain as porous as it is after printing. Third, the measuring software should be set up in a better way for this analysis; in particular, a better template size should be chosen. In our experimental setup, unfortunately, only the displacement could be evaluated without a significant error. Fourth, cameras should be better positioned to ensure measurement of displacement in the part of the sphere furthest from the rim. The main reason for preparing a better DIC measurement is obtaining a strain field from which one could derive the limit value of plastic strain (currently, the limit value is obtained from the computational model).

## 5. Conclusions

In this paper, we present that using a non-linear computational model, the stiffness can be determined with satisfactory results. The difference between modeling results and experimental data is less than 15%, which means that our simplified non-linear computational model was suitable for evaluating the stiffness properties of FS. As far as the load-bearing capacity is concerned, a full computational non-linear model was calibrated for force $F_{max}$ = 276 N which gave us limit value of plastic strain. This being said, the simplified model had an overwhelming advantage in solving time—it was approximately 160% faster than the full model; therefore, the simplified model was more useful in the parametric study. For example, the computing time for a simplified non-linear model (Specimen A1, $t_{r}$ = 0.80 mm) was 35 min. (84 iterations) on a standard machine (workstation Intel i7-8700K, 12 cores, 16 GB RAM, SSD). Approximately 25 s. was needed for each iteration. If we used a full non-linear model, the time for each iteration would be multiplied by more than 2 (i.e., 53 s. per iteration, summary 103 iterations, total time 91 min.). It must be also emphasized that the full non-linear model was not ideally converging to a solution every time. Additional tuning of the contact pair was necessary and better boundary condition needed to be set (in this case, the displacement boundary condition converged better than the force boundary condition). However, this model is highly recommended for evaluating load-bearing capacity.

Out of the three virtually tested pattern types, the pattern A performed best, mainly thanks to its high load-bearing capacity. It offered a high stiffness, which helped prevent the specimen A from changing shape under the applied load (see Figure 9). This was also confirmed in the analysis calculating the failure load. Another advantage was that the stiffness of specimen A scaled almost linearly with thickness with thickness, making it easy to design a specific stiffness value. Specimen A2 was the stiffest of the patterns A. Also, it had the highest load-bearing capacity. These properties were caused by having smaller gaps than the specimen A1. On the other hand, Specimen A3 was, compared to the other two geometries compliant, which also meant a low failure force. It was thanks to wider gaps than specimen A1. Other patterns did not offer such mechanical properties (such as stiffness and load-bearing capacity).

Our paper studied a shell, considering 2D pattern only. However, if an application needs a compliant (or rather even more compliant) structure, studying 3D-spatial pattern structures would be beneficial. The 3D-spatial structure might offer additional advantages compared to 2D structures only. For example, considering the biomechanical use, they might provide better sweat drainage and airflow, increasing the patients’ comfort. Finally, a compliant structure could also be ensured by choosing another material (one with a lower Young’s modulus than PA12), such as Thermoplastic Polyurethane (TPU).

It is necessary to mention here the cost-saving character of this virtual model. Should all configurations from Table 4 be evaluated experimentally, many more shells would have to be printed, which would immensely increase the costs. Using the presented approach, only a specific configuration is printed and evaluated experimentally, which would save roughly 90% of the costs—and this calculation is based on an experiment with a low number of specimens. To support a standard statistical evaluation, the experiment should use at least 5 to 10 specimens for each proposed structure, which would rocket the costs sky-high and the savings thanks to the use of our software solution could be over 98%.

It should be noted that the authors will continue their research on this topic and will expand on tested (2D and 3D-spatial) specimens in the following years.

## Figures and Tables

**Table 1:** Mechanical properties of PA12.

Property	Symbol	Value	Unit
Young’s modulus	*E*	1224	MPa
Poisson ratio	μ	0.39	−
Density	ρ	1010	$\mathrm{kg}m^{-3}$
Yield stress	$\sigma_{Y}$	21	MPa
Tangent modulus	$E_{T}$	334	MPa

**Table 2:** Parameters of tested structures.

Specimen	Parameter of Pattern			
Specimen A1	*b* [mm]	*l* [mm]	*p* [mm]	*t* [mm]
	2.00	5.00	2.35	1.00
				1.25
Specimen B1	*b* [mm]	*l* [mm]	-	*t* [mm]
	1.75	1.75	-	1.00
				1.25

**Table 3:** Finite element mesh statistics.

	Number of Elements	Number of Nodes
Modeling of structures stiffness	81,500	93,200
Modeling of load-bearing capacity	158,000	177,000

**Table 4:** Used specimen pattern parameters.

Pattern Parameters	Opening Parameter *b* [mm]	Opening Parameter *l* [mm]	Width between Pattern Openings *p* [mm]
Specimen A1	2.00	5.00	2.35
Specimen A2	2.00	5.00	2.60
Specimen A3	2.25	5.25	2.35
	**Opening** **Parameter ** $b_{1}$ ** [mm]**	**Width between** **Pattern Openings ** $b_{2}$ ** [mm]**	-
Specimen B1	1.75	1.75	-
Specimen B2	1.75	2.00	-
Specimen B3	2.00	1.75	-
	**Opening** **Parameter ** d ** [mm]**	**Width between** **Pattern Openings ** p ** [mm]**	-
Specimen C1	5.00	1.75	-
Specimen C2	5.00	2.00	-
Specimen C3	5.25	1.75	-

**Table 5:** Values of determined stiffness *k* from experiment.

Specimen	Designed Thickness *t* [mm]	Experiment *k* [N/mm]
A1	1.00	65.76
	1.25	81.09
B1	1.00	4.79
	1.25	7.27

**Table 6:** Comparison of determined stiffness *k* from experiment and simulation.

Specimen	Designed Thickness *t* [mm]	Real thickness $t_{r}$ [mm]	Experiment *k* [N/mm]	Simulation *k* [N/mm]	Difference [%]
Specimen A1	1.00	0.80	65.76	64.17	2.42
	1.25	1.05	81.09	86.28	6.40
Specimen B1	1.00	0.80	4.79	4.80	0.21
	1.25	1.05	7.27	6.37	12.38

**Table 7:** Evaluation of stiffness from parametric study.

Real thickness $t_{r}$ [mm]	0.80	1.05	1.30	1.55	1.80	2.05
**Flexible Structure**	Determined Stiffness k [N/mm]					
Specimen A1	64.17	86.28	110.67	132.58	152.38	170.96
Specimen A2	81.69	109.08	137.04	162.00	185.30	208.22
Specimen A3	57.52	76.40	99.11	119.04	137.04	153.74
Specimen B1	4.80	6.37	7.69	8.90	10.05	11.17
Specimen B2	5.36	7.10	8.53	9.89	11.19	12.46
Specimen B3	3.98	5.52	6.76	7.85	8.87	9.85
Specimen C1	54.21	69.93	84.87	99.66	114.30	128.80
Specimen C2	70.65	90.91	110.96	130.80	150.38	169.74
Specimen C3	47.72	62.00	75.14	88.06	100.85	113.53

**Table 8:** Percentage stiffness of A-series specimens.

Real Thickness $t_{r}$ [mm]	0.80	1.05	1.30	1.55	1.80	2.05
Flexible Structure	Percentage Stiffness [%]					
Specimen A1	100	134.5	172.5	206.6	237.5	266.4
Specimen A2	100	133.5	167.8	198.3	226.8	254.9
Specimen A3	100	132.8	172.3	206.9	238.2	267.3

**Table 9:** Comparison of failure load $F_{max}$ from experiment and simulation.

Pattern Type	Real Thickness $t_{r}$ [mm]	Experiment $F_{\max}$ [N]	Simulation $F_{\max}$ [N]	Failure Plastic Strain $\varepsilon_{\mathrm{pb}}$ [%]
Specimen A1	0.80	276	276	3.39

**Table 10:** Calculated values of load-bearing capacity.

Type of Pattern	Real Thickness $t_{r}$ [mm]	Simulation $F_{\max}$ [N]
Specimen A1	0.80	276
Specimen A2	0.80	318
Specimen A3	0.80	258

## Data Availability

Data sharing is not applicable to this article.

## Abbreviations

The following abbreviations are used in this manuscript:

FS	Flexible Structures
2D	Two-dimensional
FEM	Finite Element Method
SLS	Selective Laser Sintering
MJF	Multi-Jet Fusion
IR	Infrared
AM	Additive manufacturing
PA12	Polyamide 12
CAD	Computer Aided Design
SW	Software
FFF	Fused Filament Fabrication
PLA	Polylactic Acid
RHN	Rhinoceros
SCDM	Ansys Spaceclaim (Design Modeler)
AWB	Ansys Workbench
DIC	Digital Image Correlation
TPU	Thermoplastic Polyurethane

## References

[1] Wang S., Wang H., Ding Y., Yu F. (2020). Crushing behavior and deformation mechanism of randomly honeycomb cylindrical shell structure. Thin-Walled Struct..

[2] Paik J.K., Thayamballi A.K., Kim G.S. (1999). The strength characteristics of aluminum honeycomb sandwich panels. Thin-Walled Struct..

[3] Cheng L., Bai J., To A.C. (2019). Functionally graded lattice structure topology optimization for the design of additive manufactured components with stress constraints. Comput. Methods Appl. Mech. Eng..

[4] Marsalek P., Grygar A., Karasek T., Brzobohaty T. Virtual prototyping of 3D printed cranial orthoses by finite element analysis. Proceedings of the Central European Symposium on Thermophysics 2019 (CEST).

[5] Nagesha B., Dhinakaran V., Shree M.V., Kumar K.M., Chalawadi D., Sathish T. (2020). Review on characterization and impacts of the lattice structure in additive manufacturing. Mater. Today Proc..

[6] Alberdi R., Dingreville R., Robbins J., Walsh T., White B.C., Jared B., Boyce B.L. (2020). Multi-morphology lattices lead to improved plastic energy absorption. Mater. Des..

[7] Quoc P.M., Krzikalla D., Mesicek J., Petru J., Smiraus J., Sliva A., Poruba Z. (2020). On Aluminum Honeycomb Impact Attenuator Designs for Formula Student Competitions. Symmetry.

[8] Sotola M., Stareczek D., Rybansky D., Prokop J., Marsalek P. (2020). New Design Procedure of Transtibial Prosthesis Bed Stump Using Topological Optimization Method. Symmetry.

[9] Ligon S.C., Liska R., Stampfl J., Gurr M., Mülhaupt R. (2017). Polymers for 3D Printing and Customized Additive Manufacturing. Chem. Rev..

[10] Lee H.E., Park J.H., Jang D., Shin J.H., Im T.H., Lee J.H., Hong S.K., Wang H.S., Kwak M.S., Peddigari M. (2020). Optogenetic brain neuromodulation by stray magnetic field via flash-enhanced magneto-mechano-triboelectric nanogenerator. Nano Energy.

[11] Kim D.H., Shin H.J., Lee H., Jeong C.K., Park H., Hwang G.T., Lee H.Y., Joe D.J., Han J.H., Lee S.H. (2017). In Vivo Self-Powered Wireless Transmission Using Biocompatible Flexible Energy Harvesters. Adv. Funct. Mater..

[12] Schumacher C., Marschner S., Gross M., Thomaszewski B. (2018). Mechanical characterization of structured sheet materials. ACM Trans. Graph..

[13] Bickel B., Bächer M., Otaduy M.A., Lee H.R., Pfister H., Gross M., Matusik W. (2010). Design and fabrication of materials with desired deformation behavior. ACM Trans. Graph. (TOG).

[14] Schumacher C., Thomaszewski B., Gross M. (2016). Stenciling. Comput. Graph. Forum.

[15] Hussain G., Khan W.A., Ashraf H.A., Ahmad H., Ahmed H., Imran A., Ahmad I., Rehman K., Abbas G. (2019). Design and development of a lightweight SLS 3D printer with a controlled heating mechanism. Int. J. Lightweight Mater. Manuf..

[16] Cai C., Tey W.S., Chen J., Zhu W., Liu X., Liu T., Zhao L., Zhou K. (2021). Comparative study on 3D printing of polyamide 12 by selective laser sintering and multi jet fusion. J. Mater. Process. Technol..

[17] Řepa V., Maršálek P., Prokop J., Rybanský D., Halama R. (2020). Modelling and Testing of 3D Printed Flexible Structures with Three-pointed Star Pattern Used in Biomedical Applications. Experimental Stress Analysis 2020.

[18] Faustini M.C., Neptune R.R., Crawford R.H., Rogers W.E., Bosker G. (2006). An Experimental and Theoretical Framework for Manufacturing Prosthetic Sockets for Transtibial Amputees. IEEE Trans. Neural Syst. Rehabil. Eng..

[19] Lammens N., De Baere I., Van Paepegem W., Ragaert K., Delva L., Cardon L. (2016). On the orthotropic elasto-plastic material response of additively manufactured polyamide 12. Proceedings of the 7th Bi-Annual International Conference of Polymers & Moulds Innovations.

[20] Stoia D., Linul E., Marsavina L. (2019). Influence of Manufacturing Parameters on Mechanical Properties of Porous Materials by Selective Laser Sintering. Materials.

[21] Kminek T., Marsalek P., Karasek T. (2019). Analysis of steel tanks for water storage using shell elements. AIP Conference Proceedings.

[22] Lesnak M., Marsalek P., Horyl P., Pistora J. (2020). Load-bearing capacity modelling and testing of single-stranded wire rope. Acta Montan. Slovaca.

[23] Horyl P., Snuparek R., Marsalek P., Poruba Z., Paczesniowski K. (2019). Parametric studies of total load-bearing capacity of steel arch supports. Acta Montanistica Slovaca.

[24] Bai J., Song J., Wei J. (2019). Tribological and mechanical properties of MoS2 enhanced polyamide 12 for selective laser sintering. J. Mater. Process. Technol..

[25] Dorčiak F., Vaško M., Handrik M., Bárnik F., Majko J. (2019). Tensile test for specimen with different size and shape of inner structures created by 3D printing. Transp. Res. Procedia.

[26] Dorčiak F., Vaško M., Bárnik F., Majko J. (2020). Comparison of experimental flexural test with FE analysis for specimen with different size and shape of internal structure created by 3D printing’s. IOP Conf. Ser. Mater. Sci. Eng..

[27] Halama R., Pagáč M., Paška Z., Pavlíček P., Chen X. Ratcheting Behaviour of 3D Printed and Conventionally Produced SS316L Material. Proceedings of the ASME 2019 Pressure Vessels & Piping Conference.

